# The Role of Mesenchymal Stem Cells for Corneal Endothelial Regeneration: A Systematic Review

**DOI:** 10.5041/RMMJ.10531

**Published:** 2024-10-28

**Authors:** Siska Siska, I. Gede Eka Wiratnaya, I. Made Bakta, I. Made Jawi, I. Gde Raka Widiana, Putu Yuliawati, Made Ratna Saraswati, Heri Suroto

**Affiliations:** 1 Department of Ophthalmology, Faculty of Medicine, Udayana University, Denpasar, Indonesia; 2 Department of Orthopedics and Traumatology, Faculty of Medicine, Udayana University, Denpasar, Indonesia; 3 Department of Internal Medicine, Faculty of Medicine, Udayana University, Denpasar, Indonesia; 4 Department of Pharmacology, Faculty of Medicine, Udayana University, Denpasar, Indonesia; 5 Department of Orthopaedics and Traumatology, Faculty of Medicine, Airlangga University, Surabaya, Indonesia

**Keywords:** Anterior chamber, corneal endothelial cell density, corneal endothelial regeneration, *in vivo*, mesenchymal stem cells

## Abstract

**Objective:**

A single layer of tightly spaced cells, known as the endothelium, rests on the posterior side of the cornea. This endothelium regulates the stroma’s relative dehydration, which is essential for corneal clarity. Cell therapy is an innovative method being used to repair various corneal abnormalities. Mesenchymal stem cells (MSCs) are now one of the most significant types of stem cells scientists have studied. This study aimed to evaluate the role of MSCs for corneal endothelial regeneration.

**Methods:**

A systematic review was performed by searching for articles from reputable databases with many study-type references, including PubMed, Cochrane Library, Science Direct, and Google Scholar, up to January 2024. The resulting data were displayed using the 2020 PRISMA flowchart and evaluated using the PRISMA 2020 checklist. Most of the included studies were *in vivo* and used topical application and anterior chamber injection as the administration routes.

**Results:**

Based on the findings of this review, MSCs increased corneal endothelial cell density, improved the defect area and corneal transparency, facilitated endothelial cell regeneration and wound healing, and decreased neovascularization and corneal pro-inflammatory cytokines as compared to controls.

**Conclusion:**

Administration of MSCs into the anterior chamber could increase regeneration and proliferation of corneal endothelial tissue.

## INTRODUCTION

The cornea is the clear aperture that allows light to enter the eye. In adults, the diameter and thickness of this avascular tissue measure 10–12 mm and 500–600 μm, respectively, with a light-refractive index of 1.38. The outer layer of the cornea consists of stratified corneal epithelial cells. A monolayer of densely packed, hexagon-shaped corneal endothelial cells (CECs) lines the innermost layer of the cornea. The CECs reside in contact with the stroma on the Descemet membrane.^1^

On the posterior side of the cornea, a single layer of tightly spaced cells called the endothelium regulates the stroma’s relative dehydration, which is essential for corneal clarity.^2^ A corneal endothelium with adequate cell density maintains normal corneal moisture and transparency by acting as both a passive barrier and an active ion transporter. Endothelial cell density diminishes with age steadily and linearly. However, more rapid reduction in cell density may occur in response to surgical or accidental trauma, corneal transplantation, and illnesses, including endothelial dystrophy, glaucoma, and diabetes.^3^

Corneal endothelial decompensation occurs when the density of endothelial cells in the cornea drops below a critical level.^4^ The only known treatment is to transplant a donor cornea with a healthy endothelium.^5^ Human CECs are not physically capable of regenerating *in vivo*. Cell migration and expansion in the defect area facilitate endothelial wound repair.^6^ On the other hand, *ex vivo* studies have revealed that the periphery of the corneal endothelium has a greater capacity for *in vitro* regeneration than the center.^7^

One of the leading causes of blindness worldwide is corneal damage. Corneal transplantation is a way to treat corneal damage. However, approximately 12.7 million people globally are in need of corneal transplants.^8^ Among the difficulties and obstacles associated with corneal transplantation are its high cost, the risk of transplant rejection, the scarcity of donors, as well as legal and cultural concerns.^9^ Cell therapy is an innovative method for repairing various corneal abnormalities. One of the newer cell therapies is related to stem cells. Tissue engineering uses adult and embryonic stem cells to repair damaged organs and tissues because of their capacity for both differentiation and reproduction. Mesenchymal stem cells (MSCs) are now one of the most significant types of stem cells studied by scientists.^10^ This study aimed to evaluate the role of MSCs in corneal endothelial regeneration.

## MATERIALS AND METHODS

### Literature Review Criteria

For the purposes of this review, “corneal endothelium” refers to the monolayer of hexagon-shaped cells that line the posterior surface of the cornea and divides the aqueous humor of the anterior chamber from the corneal stroma. This review included all animal model studies that used an MSC-related intervention in corneal endothelial tissue.

Each study considered for inclusion had to assess the effects of MSCs on injured rabbit corneal endothelium using a controlled methodology. Therefore, this review included only studies where MSC-related therapy was the main element of the intervention being assessed. The inclusion criteria were as follows: (1) *in vivo* animal studies and human trials, including both small animals (mice, rats, rabbits, and hamsters) and large animals (pigs, dogs, and horses), standardized according to the appropriate animal laboratory protocols; (2) studies using models of damaged corneal endothelium; (3) interventions involving human-derived stem cells injected or transplanted into specified or defective sites; and (4) studies published in the last 10 years (2013–2023) in internationally recognized journals. The exclusion criteria were: (1) *in vitro* animal studies; (2) interventions using only non-human-derived stem cells; and (3) studies using mixed interventions with additional treatments such as specified growth factors.

Objective and self-reported outcome measures (Table 1) were retrieved from studies that met the inclusion requirements.

**Table 1:** Outcome Measures.

Parameter for MSC-based Intervention	Outcome Measures	
	Objective Outcomes	Subjective Outcomes
Tissue regeneration	Cytokine and growth factor (e.g. FGF, TGF-β, and IL-1) levels Endothelial cell count Corneal thickness	Cornea clarity
Side effects and complication	Corneal edema	Cornea clarity (transparency) Visual function

FGF, fibroblast growth factor; IL-1, interleukin-1; TGF-β, transforming growth factor beta.

### Literature Search

The authors used the Population, Intervention, Comparison, Outcome, Studies (PICOS) criteria listed in Table 2 to identify relevant articles. All data taken from systematic searches were recorded in the 2020 PRISMA flowchart and evaluated using the PRISMA 2020 checklist.

**Table 2:** PICOS Strategies.

Criteria	Definitions
Population	Damaged corneal endothelial animal model
Intervention	MSCs
Comparator	Any comparator
Primary outcomes	Between-group level differences of TGF-β, and IL-1, endothelial cell count and corneal thickness
Secondary outcomes	Between-group level differences of FGF, endothelial cell count, and corneal thickness
Study design	Original animal experimental studies

FGF, fibroblast growth factor; IL-1, interleukin-1; MSCs, mesenchymal stem cells; PICOS, population, intervention, comparison, outcome, studies; TGF-β, transforming growth factor beta.

The data used in this review were secondary data obtained from included studies. Literature searches were performed using controlled vocabulary based on the PICOS framework to get general search terms (keywords). After selecting keywords and their synonyms using Medical Subject Headings (MeSH), searches of research journals were performed using advanced search, bibliographic searching, and Boolean operators (AND, OR, and NOT) on keywords arranged according to the research topic. The keywords and Boolean operators used were: (“mesenchymal stem cells” OR “mesenchymal stem cell” OR “mesenchymal”) AND (“corneal endothelial cell” OR “corneal regeneration”) AND (“animal model”).

Searches were performed for all dates up to January 2024 using the following reputable databases: PubMed, Cochrane Library, Science Direct, and Google Scholar. All relevant studies that met the inclusion criteria, but not the exclusion criteria, were used in this review.

### Data Extraction

Following a full-text analysis, the following data were collected from the included articles: title, author details, publication year, evaluated outcome(s), kind of animal model, animal weight, age, and sex, dosage administered, duration of research, mode of administration, and primary findings.

A synthesis matrix was used to collate all findings, together with each article’s weaknesses and strengths, enabling presentation of the data in a structured manner. After reviewing the data, descriptions and conclusions could be made regarding the similarities and differences among the findings. The research results were then reviewed again with a supervisor and examiner to ensure more precise, accurate, consistent, and unambiguous analyses and presentation of the data.^11^

### Assessing Quality and Risk of Bias

Risk of bias and quality of the animal studies were evaluated using the Systematic Review Centre for Laboratory Animal Experimentation (SYRCLE) Risk of Bias instrument. The SYRCLE technique is a modified version of the Risk of Bias tool, made available by the Cochrane Collaboration, and intended to translate risk assessments from human research to animal studies. The SYRCLE tool comprises 10 items that are linked to biases in reporting, attrition, detection, performance, and selection. Overall bias is divided into low risk of bias, unclear risk of bias, and high risk of bias based on assessor assessments in five domains.^12^ The risk of bias for each study was thoroughly evaluated by two researchers. In case of disagreements among the researchers, an impartial third researcher made the final decision. Relevant studies were eliminated from the study if the estimated risk of bias was high.

### Data Analysis

Data analysis was performed by systematically integrating and describing all data to obtain conclusions. The data consisted of study characteristics (name of primary author, year of publication, and research location), participant characteristics (animal type, age, and weight), and pre-determined outcomes. All data from the analysis are presented in Table 3.

**Table 3:** Characteristics of the Studies.

Author (Year)^ref.^	Country	Study Design	Animal Model	Animal Sex	Age	Weight	Dose Administered	Administration Route	Study Length
Ali et al. (2021)^13^	USA	*In vivo*	NZ and Mky	NZ: M and F Mky: M	NZ: 12–14 wk Mky: 14 y	NZ: 2.5–3.5 kg Mky: 14 kg	NZ: 7.5x10^5^ cryopreserved hESC-derived endothelial-mesenchymal transformed CECs Mky: 1.0x10^6^ cryopreserved hESCs	Injected into the anterior chamber	9–21 mo
Alio del Barrio et al. (2015)^14^	Spain	*In vivo*	NZ	NR	NR	NR	2x10^5^ cells in 500 mL	Injected into the implanted sheet	12 wk
Damala et al. (2023)^15^	India	*In vivo*	C57BL/6 mice	NR	6–8 wk	20–25 g	5x10^4^ En−/En+ hLMSCs mixed in 2 μL of fibrin glue	Topical	4 wk
Demirayak et al. (2016)^16^	Turkey	*In vivo*	Wistar rat	F	6 wk	180–220 g	2x10^5^ cells	Injected into the anterior chamber	8 wk
Di et al. (2017)^17^	China	*In vivo*	C57BL/6 mice	M	6–8 wk	NR	5x10^4^ MSCs	Subconjunctival injection	48 h
Nieto-Nicolau et al. (2021)^18^	Spain	*In vivo*	Wistar rat	NR	NR	250–300 g	Amniotic membrane ocular surface implants with adipose tissue-derived MSCs; no specific doses stated	Topical	30 d
Ryu et al. (2023)^19^	South Korea	*In vivo*	Sprague Dawley rats	F	6 wk	NR	4.5x10^8^/10 μL	Injected into the anterior chamber	2 wk
Saccu et al. (2022)^20^	Italy	*In vivo*	FVB mice	F	3 mo	NR	Extracellular vesicles derived from bone marrow MSCs/stromal cells and embedded in methylcellulose	Topical	2 wk
Sendon-Lago et al. (2019)^21^	Spain	*In vitro* and *in vivo*	NZ	F	NR	2–2.5 kg	Lyophilized CM-hUCESC suspended in 1.25 mL of ddH_2_O given four times per day, for a total duration of 60 hours	Topical	60 h
Shukla et al. (2019)^22^	USA	*In vivo*	C57BL/6J mice	M and F	6–8 wk	NR	5×10^5^ cells	Topically and via subconjunctival injection	4 d
Sun et al. (2017)^23^	China	*In vitro* and *in vivo*	NZ	NR	NR	2.0–4.0 kg	Carboxyfluorescein succinimidyl ester-labeled HCECs (3.0x10^5^ cells) suspended in 100 μL opti-minimal essential medium to induce EMT	Injected into the anterior chamber	2 mo
Then et al. (2017)^24^	Malaysia	*In vivo*	NZ	Male	Adult	1.8–2.3 kg	–	Transplantation	2 mo
Ye et al. (2022)^25^	South Korea	*In vitro* and *in vivo*	NZ	NR	NR	1.8–2.2 kg	4.1x10^6^ MSCs-derived circulating endothelial cells in 150 μL of PBS supplemented with 100 μM of Y-27632	Injected into the anterior chamber	3 wk

CECs, corneal endothelial cells; CM-hUCESC, conditioned medium from human uterine cervical stem cells; d, day(s); ddH_2_O, double-distilled water; EMT, endothelial-to-mesenchymal transition; F, female; FVB, Friend Virus B; HCECs, human corneal endothelial cells; hESCs, human embryonic stem cells; hLMSCs, human limbus-derived stromal/mesenchymal stem cells; M, male; Mky, monkeys; mo, month(s); MSCs, mesenchymal stem cells; NZ, New Zealand white rabbits; NR, not reported; PBS, phosphate-buffered saline; wk, week(s); y, year(s).

## RESULTS

**Figure 1:**
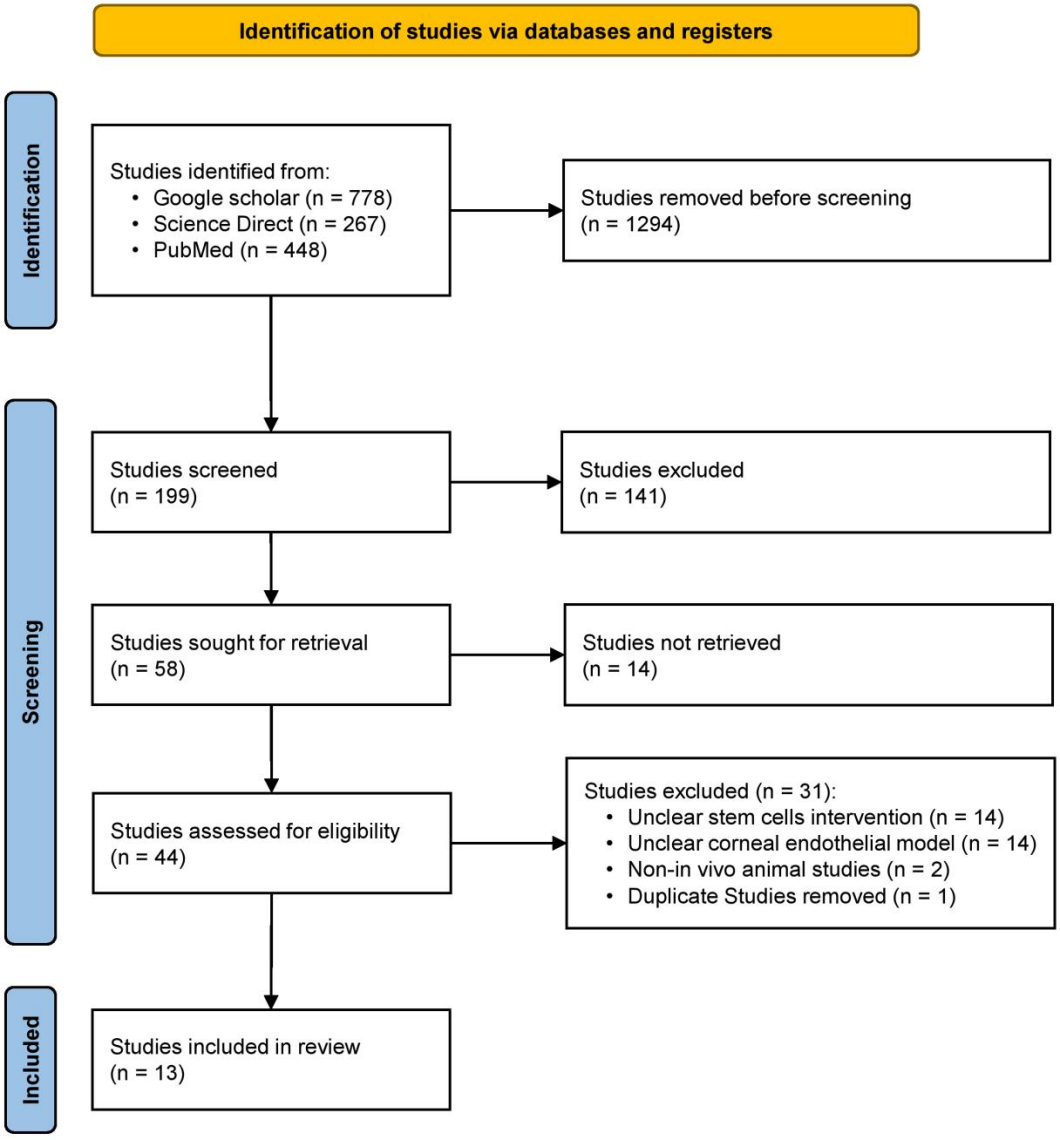
PRISMA Flowchart.

The literature search found a total of 1,493 studies relevant to this review. Referring to the PRISMA flowchart (Figure 1), 1,294 records were excluded prior to screening in line with the inclusion and exclusion criteria specified by the tools in each database. A total of 141 studies were disqualified after reviewing the title and abstract, 14 studies could not be retrieved, and 31 studies were excluded due to unclear stem cell intervention, unclear corneal endothelial model, and non-*in vivo* animal studies.

A total of 13 studies were included in this review.^13^–^25^ Based on the SYRCLE Risk of Bias instrument, all 13 studies had a low risk of bias.

Of the 13 included studies, 10 were *in vivo* studies, and three were combined *in vivo* and *in vitro* studies. No human trials were retrieved via the literature search process. Of the 13 studies, 3 were from Spain; 2 each were from China, South Korea, and USA; and 1 each from India, Italy, Malaysia, and Turkey. Most administration routes included in the studies were topical application and anterior chamber injection. The duration of the studies varied from 48 hours to several months. Table 3 provides an overview of the included studies.

### Study Outcomes

The studies reported that MSCs increased CEC density, improved the defect area and corneal transparency, facilitated endothelial cell regeneration and wound healing, and decreased neovascularization and corneal pro-inflammatory cytokines compared to the control. The study outcomes are provided in Table 4.

**Table 4:** Study Outcomes.

Author (Year)	Assessed Outcome	Main Result
Ali et al. (2021)^13^	Efficacy of cryopreserved human embryonic stem cell-derived CECs to form a functional monolayer of corneal endothelium	CEC density of injected eye was >80% of the CEC density of the untreated eye Central corneal thickness of the injected eye remained comparable (±20 mm) to the untreated eye
Alio del Barrio et al. (2015)^14^	Biocompatibility of grafts composed of sheets of decellularized human corneal stroma with or without the recellularization of human adipose-derived adult stem cells into the rabbit cornea	The hypocellular band was observed, containing cells with stellate morphology around and inside the sheet in the treated group
Damala et al. (2023)^15^	Effectiveness in stopping corneal scar development and corneal surface regeneration	In every treatment arm, the damaged corneal surface area progressively shrank
Demirayak et al. (2016)^16^	Corneal scarring after penetrating injury	Significant difference in the mean anterior keratocyte density and mean posterior keratocyte density values of the transplanted groups versus the control group
Di et al. (2017)^17^	Diabetic corneal epithelial wound healing	Defect area of corneal epithelium in MSC-treated diabetic mice significantly improved compared to untreated diabetic mice
Nieto-Nicolau et al. (2021)^18^	Human AT-MSCs for corneal surface regeneration	AT-MSCs cultured with standard medium improved corneal transparency and decreased neovascularization in comparison with non-treated and amniotic membrane-treated groups
Ryu et al. (2023)^19^	Corneal endothelial cells proliferation	Exosomes generated from AT-MSCs facilitated endothelial cell regeneration and wound healing by causing a change in the cell cycle and inhibiting autophagy and senescence
Saccu et al. (2022)^20^	Corneal repair through histological and molecular analyses	Bone marrow-derived-MSC-derived extracellular vesicle formulation significantly accelerated corneal repair by modulating cell death, inflammation, and angiogenesis in a murine model of alkali-burn-induced corneal damage; similar effects observed *in vitro* on human corneal epithelial cells
Sendon-Lago et al. (2019)^21^	The effect and mechanism of action of the CM-hUCESC on corneal wound healing	CM-hUCESC induces faster corneal regeneration in a rabbit atropin-induced dry eye model and reduces corneal pro-inflammatory cytokines
Shukla et al. (2019)^22^	The efficacy of MSC administration in corneal injury	MSCs significantly suppressed injury-induced corneal opacification
Sun et al. (2017)^23^	The expansion and function of HCECs	High expression levels of vimentin, CD29, CD105, CD49e, and CD166 noted in cultured human OASCs Expression of CEC-related markers zonula occludens-1 (ZO-1), Na+/K+ ATPase, N-cadherin, Col8a2, and SLC4A4 in OASC-CM-cultivated HCECs The HCECs maintained their excellent proliferative ability and polygonal cell shape Corneal transparency achieved in animals after HCEC-injection
Then et al. (2017)^24^	The effectiveness of treating corneal stromal deficiency using autologous MSCs generated from bone marrow	Localization of PKH26-labeled BM-MSCs revealed increased cell density in the transplanted location, indicating a role in corneal stromal regeneration
Ye et al. (2022)^25^	The efficacy of anterior chamber injection of MSC-induced CECs	Human umbilical cord-derived MSCs were successfully differentiated into CECs in vitro; injection into a rabbit model of CED improved corneal opacity and neovascularization

AT-MSCs, adipose tissue-derived mesenchymal stem cells; BM-MSCs, bone marrow mesenchymal stem cells; CEC(s), corneal endothelial cell(s); CED, corneal endothelial dysfunction; CM-hUCESC, conditioned medium from human uterine cervical stem cells; HCECs, human corneal endothelial cells; MSC(s), mesenchymal stem cell(s); OASCs, orbital adipose-derived stem cells; OASC-CM, orbital adipose-derived stem cells conditioned medium.

## DISCUSSION

This literature review has shown that MSCs have the potential to regenerate CECs. Depending on their origin, stem cells may be classified as adult or embryonic stem cells and have the ability to develop into MSCs.^8^ They interact with both innate and adaptive immune cells and are essential for regulating immune responses via paracrine signaling.^26^ The role of MSCs vary, depending on their source: both bone marrow and adipose MSCs exhibit the phenotypic markers CD13, CD73, CD90, CD105, and STRO-1; however, their CD34, CD49d, CD54, and CD106 expression patterns are distinct. Compared to MSCs from birth-associated tissues, MSCs obtained from adult tissues such as bone marrow have lower proliferation, engraftment ability, and differential potential.^8^ In addition, MSCs can also have molecular derivatives, namely exosomes that function in paracrine signaling between cells directly. Exosomes play an important role in intercellular communication, which affects the cellular environment; in the context of corneal therapy, exosomes help regulate tissue inflammatory responses, modulate cytokine and chemokine balance, and improve epithelial wound healing.^27^

Because MSCs can control immune responses, they are more effective as therapeutics and less likely to be rejected in both the *in vitro* and *in vivo* environments. Believed to be immune-privileged cells, the cell surfaces of MSCs have fewer major histocompatibility complex class II molecules and co-stimulatory molecules (CD80, CD86, and CD40). Mesenchymal stem cells influence the innate immune system *in vitro* by inhibiting the cytotoxicity of natural killer cells and developing and activating dendritic cells. Additionally, they impede the maturation of B and T cells and their ability to proliferate and secrete cytokines, thereby suppressing adaptive immunological responses.^28^ In addition, scientists are investigating in-cell treatment as a potential substitute for corneal transplantation, which is sometimes hindered by a lack of donors. It has been shown that corneal endothelium transplantation is a feasible alternative since rho-associated protein kinase (ROCK) can renew and repair CECs.^8^

The rho protein is an essential regulator of skeletal structure. It is hypothesized that ROCK signaling inhibition may influence cell adhesion characteristics, and CEC transplantation may be used as a therapeutic intervention in regenerative medicine. Corneal transparency was enhanced in a rabbit with endothelial failure by transplanting CECs with a ROCK inhibitor. This approach may be used in human clinical trials despite using an animal model for this work.^10^ Using Descement membrane biomimetic microphotography, human MSCs may be induced to develop into corneal endothelial-like cells. The ZO-1 and Na/K-ATPase proteins and *COL81*, *COL8A2*, and *PITX2* genes are exclusive to the endothelium and may all be expressed by MSCs.^29^

Mesenchymal stem cells derived from the lining of the umbilical cord and bone marrow can differentiate into keratocyte-like cells and may be able to reinstate transparency to the corneal stroma. Injecting human umbilical cord lining MSCs into the corneal stroma improved the aberrant collagen structure, restored corneal thickness, and enhanced corneal transparency. Furthermore, there was little chance of rejection since the injected cells reduced the inflammatory cytokine levels.^30^

### Limitations

A limitation of this review is that it only included English-language literature; hence, some of the unincluded studies may have had skewed data. Also, since use of MSCs is a contentious strategy, no studies could be located that used human models.

### Future Research

Although MSC therapy holds promising potential in treating corneal tissue-related diseases, several challenges must be addressed before widespread clinical application can be achieved. First, there is currently no standardized protocol for isolating and characterizing MSCs across studies, making it difficult to conduct valid comparative analyses of MSCs treatment efficacy. Therefore, future research should focus on establishing standards for MSCs isolation and characterization, including determining effective administration methods, such as intrastromal, subconjunctival, and topical approaches.

Second, the challenge of maintaining the survival of transplanted MSCs in corneal tissue remains significant. While several studies reviewed here reported favorable long-term outcomes, the results varied considerably. In consequence, future research should also focus on strategies to enhance long-term MSCs survival, such as genetic modification or preconditioning.

Third, *in vivo* efficacy measurements of MSCs, through to clinical trials, are still limited. This may be due to resource constraints and limited patient availability, which could impact the findings on the safety and efficacy profiles of the therapy. Nonetheless, these challenges are likely surmountable, allowing the continued development of MSC therapy to show increasingly promising results.

## CONCLUSION

In conclusion, the administration of MSCs both topically and by anterior chamber injection could increase regeneration and proliferation of corneal endothelial tissue in animal models based on histological and molecular findings (cytokine and growth factors). Further research is needed to evaluate the application of human-derived stem cells in both animal and human populations.

## Abbreviations

CEC(s): corneal endothelial cell(s)
MeSH: medical subject headings
MSC(s): mesenchymal stem cell(s)
PICOS: population, intervention, comparison, outcome, studies
ROCK: rho-associated protein kinase
SYRCLE: Systematic Review Centre for Laboratory Animal Experimentation.
